# Design model for inclusion of resilience in children with cerebral palsy

**DOI:** 10.4102/ajod.v14i0.1715

**Published:** 2025-10-08

**Authors:** Aditya D. Pratama, Rachmadhi Purwana, Jan Sopaheluwakan, Diaz Pranita, Bintang M.B. Akbar

**Affiliations:** 1 School of Environmental Sciences, Universitas Indonesia, Jakarta, Indonesia; 2 Vocational Education Program, Universitas Indonesia, Depok, Indonesia

**Keywords:** cerebral palsy, disability, inclusion, resilience, sustainability

## Abstract

**Background:**

Regarding the continuity of life, people with disabilities require various forms of assistance to fulfill their rights to survive and be sustainable. Children with cerebral palsy (CP) disabilities have not yet had their social, economic and environmental rights fulfilled as people with disabilities. This condition drives the need to create an inclusion model for the resilience of children with CP disabilities to provide a competitive advantage in quality and empowered human resources.

**Objectives:**

This study aimed to explore the important factors and strategies for enhancing the resilience of children with CP.

**Method:**

The data used in this study are based on the Penta helix (government, community, practitioners, society and academics). The soft system method was chosen to analyse the study’s problems.

**Results:**

Inclusion for resilience in children with CP disabilities has excellent potential to be optimised by managing several important factors, including policies, databases, health, social aspects, infrastructure and education.

**Conclusion:**

This can be achieved through genuine collaboration among key stakeholders: the government, society, medical professionals, communities and academics. With genuine collaboration and implementation, the target of resilience inclusion for children with CP will be achieved with good physical and mental health and quality of life.

**Contribution:**

This study provides new and more comprehensive knowledge about the key factors and strategies to enhance the resilience of children with cerebral palsy disabilities.

## Introduction

In the modern world, the health issues of persons with disabilities rarely receive adequate attention from the government, businesses and society in general (Wiryawan 2022). People who have experienced physical, mental, intellectual or sensory limitations for a long time and have faced various challenges may find it difficult to fully participate and succeed in a society where everyone has equal rights (Widhawati, Santoso & Apsari 2020). There are more than 1 billion people with disabilities worldwide, with 82% of them living in developing countries, living in poor economic conditions, facing high levels of violence and belonging to the most marginalised groups (Rifai & Humaedi 2020). More than 37 million persons with disabilities live in Indonesia, with 17 million of them being children and adults (United Nations 2020). People with disabilities are vulnerable to challenges and issues that limit their fulfilment of life’s rights. In terms of life sustainability, individuals with disabilities require various forms of assistance to fulfil their rights so that they can survive and be sustainable (Figueiredo et al. 2023).

As one of the countries that has adopted the Convention on the Rights of Persons with Disabilities (CRPD), Indonesia is committed to realising the rights of persons with disabilities. This is regulated in Article 3, Paragraph 1 of Law No. 8 of 2016 Concerning Disabilities, Implementation, and Fulfillment of the Rights of Persons with Disabilities. The purpose of this law is to realise respect, promotion, protection and fulfilment of human rights, as well as equal basic freedoms for persons with disabilities. Previous research results still overlook and have not yet addressed a more holistic problem-solving approach involving pressure and release or reversal actions to eliminate, reduce, avoid and resolve the present root problems (Chen et al. 2014; Jawed & Mowry 2023; Noten et al. 2021; Ungar et al. 2007). Additionally, a socio-demographic approach from each country is needed. Therefore, further research with a sociodemographic approach and appropriate modelling methods is urgently needed. In the end, the development of a resilience inclusion (systems and policies that not only ensure equal participation but also build community capacity to survive and thrive in the face of challenges, whether social, economic or environmental) model for children with cerebral palsy (CP) disabilities will be one of the steps to enhance the resilience of children with CP disabilities. All these issues have driven the need to develop a specific model to provide insight into the inclusion of resilience for children with CP disabilities. The model is a comprehensive study of CP disabilities by examining factors included in the pressure model (root cause, dynamic pressure and unsafe condition) and the release model (addressing root causes, reducing pressure and achieving safe conditions) from social, economic and environmental aspects and involving individual aspects as an integral part of problem solving (Wisner et al. 2004). Essentially, the model aims to provide a comprehensive understanding of CP disability through two primary approaches: the stress model and the release model. The stress model identifies factors that exacerbate CP, including medical root causes, social and economic pressures and unsupportive conditions. Meanwhile, the release model focuses on solutions to address the root causes, reduce pressure and create a safe and supportive environment for patients with CP. This approach can reduce the impact of disability on individuals with CP through holistic changes in medical, social and environmental aspects.

### Theoretical framework

The theoretical framework in this study uses the pressure and release model approach, which is one of the appropriate approaches for persons with CP. Based on the literature review and information collection, a comprehensive person with CP model that identifies and analyses factors related to the pressure model (root cause, dynamic pressure and unsafe condition) and the release model (addressing root causes, reducing pressure and achieving safe conditions) has not been performed. Studies on existing CP issues include the following: (1) Ungar et al.’s (2007) Resilience Model, which describes resilience resources in seven components. This model has not yet explored and linked the existing categories of resources. In addition, a priority scale based on the root causes and problem priorities has not yet been created. (2) The International Classification of Functioning, Disability and Health (ICF) (Chen et al. 2014) model evaluates the determinants related to children’s activities, followed by body functions, structures and family factors. However, it does not evaluate factors related to participation and the children themselves. (3) The International Classification of Functioning, ICF model (Noten et al. 2021) is viewed from the framework of the World Health Organization’s ICF, which serves as the basis for global health communication. However, this model focuses only on activities, body functions and structures. This model only represents adult CP disabilities; therefore, risk mitigation and related transitions during childhood over time and participation need to be considered. This model has not yet considered each country’s social and cultural conditions and socio-demographic characteristics, especially Indonesia. In addition, there is a lack of participant involvement and facilitation of individual and collective decision-making. (4) The American Academy of Pediatrics (AAP) model, in the 2022 clinical practice guidelines for CP (Jawed & Mowry 2023), includes various considerations aligned with the biopsychosocial model, risk and resilience, as well as family-centered services, and promotes a more strength-based approach in care. This model has not yet considered operational considerations (e.g. finances and staffing), funding and community engagement in ensuring sustainable access to timely and necessary services for patients with CP from the onset of diagnosis as a measure of sustainability in CP care. Although this model is quite good, it still neglects and does not consider a more holistic approach to problem-solving through pressure and release and/or reversal actions to eliminate, reduce, avoid and mitigate the root causes of existing issues. Additionally, a socio-demographic approach from each country is needed. This study focuses on Indonesia, specifically in Jakarta Province, by analysing the region’s context and relevant issues.

Suppose the death of a person with CP disabilities is considered a disaster. In that case, this research aims to build a disability inclusion model through a risk mitigation perspective that is expected to minimise the risk of hazards that will occur and minimise hazards such as reducing the physical capacity of CP, declining health conditions of CP and even the worst case, death.

## Research methods and design

### Study design

This research designs a model to provide a foundation for developing resilience inclusion for children with CP through a qualitative approach.

### Study population and sampling strategy

The primary data in this study refer to the penta helix model involving five entities: (1) Government, (2) Practitioners, (3) Academics, (4) Society, and (5) Community. Three individuals represent each expert group.

### Data collection

Data were obtained through intensive interviews supported by literature studies from appropriate and credible sources and through the distribution of closed and open questionnaires. The source has approved the data obtained to be used as data in this research.

### Data analysis

The analysis method used in this study is the Soft System Methodology (SSM). Williams (2005) explains that SSM emphasises solving something complex by providing explanations and synthesis to get the best answer. Zarei, Azizian and Ghapanchi (2013) emphasised the use of SSM to build and develop models. Daellebach and McNickle (2005) explained that SSM is the proper method for comprehensively explaining the conditions and development plans of the objects studied. Soft Systems Methodology is an approach to solving complex and unstructured problems by involving stakeholders in the problem-solving process. A quantitative approach is not necessary because SSM is sufficient to provide a deep understanding of complex problems. Soft Systems Methodology emphasises holistic system analysis by considering qualitative aspects, such as the views and perceptions of various actors within the system and the interactions between the elements involved in the social, cultural and organisational context. The SSM approach involves problem identification, system model development, stakeholder discussions, solution evaluation and implementation to solve complex problems by considering various social and human perspectives.

### Ethical considerations

Ethical approval for conducting this research was obtained from the Ethics Review Committee of the School of Environmental Science, Universitas Indonesia on 02 August 2024 (No. KET-081/UN2.F13.D1.KE1/PPM.00/2024).

## Results and discussion

The soft system technique stages outlined by Checkland and Scholes (1991) encompasses seven distinct phases. The initial phase completely and systematically outlines the challenges encountered; information is gathered from stakeholders within the inclusion of resilience in children with CP throughout this phase. The second phase delineates the problem’s context with the data acquired from the previous stage. The outcome of this phase is the formulation of a detailed depiction of the current circumstances within the item. The third phase involves identifying the issues encountered by the study subject. The foundation for this identification, using the P strategy, is conducted through Q to obtain R. In the fourth phase, the Customers, Actors, Transformation, Worldview, Owners, and Environmental constraints (CATWOE) describe and connect several important parts that can be involved. In the next phase, the built design is compared with the actual design. The comparison activity is intended to determine the possibility of maintenance, repair or evaluation. The initial stage of the SSM involves the description of the problem scenario. At this initial stage, social analysis is conducted to examine the suitability, patterns, interests and logic of relevant and appropriate events (Rouse & Daellebach 2002).

This phase emphasises understanding each informant’s circumstances and interests (Fitriati 2015). Hardjosoekarto (2012) explained that the initial stage emphasises actual social conditions as the basis for the change plan. Standardisation is carried out by categorising the obtained data. Checkland and Poulter (2006) explained that three aspects need to be explored, namely, norms, roles and values as a fundamental basis. The basic concept of standards for evaluating each stakeholder’s role is called value. Norms refer to the behaviour expected of actors in their roles, whereas roles represent the social position that characterises individual actors.

Based on social analysis, several conflicts hinder the process of inclusive development for children with CP disabilities, including the following:

- The lack of synergy among government agencies persists because the inclusive development process remains hindered. This condition is attributed to suboptimal coordination among relevant government agencies, including the Ministry of Health, the Ministry of Education and other social institutions. The lack of policy alignment between agencies results in fragmented and less practical inclusive services provided to children with disabilities, especially those with CP.
- Lack of inclusive medical and educational personnel affects the quality of medical care and the educational process that can optimally support the development of children with CP. The addition of experts with specialised skills in handling children with special needs is essential.
- The current inclusive policies are not comprehensive and detailed enough to fully accommodate all aspects required by children with disabilities. More specific and comprehensive policies are needed to support the development of inclusion, including the provision of accessible facilities, strengthening the inclusive education system and broader social support.
- Social movements that support the inclusion of children with disabilities must be strengthened to encourage policy changes and their implementation. Policy advocacy communities must provide strong encouragement so that the inclusion process can be effectively implemented. This movement must involve various parties, including the community, non-governmental organisations and the government sector, to advocate for the rights of children with disabilities.
- Aligning the understanding of disability among all parties, including the community, medical professionals, educators and policymakers, is a crucial factor in developing inclusive practices. Uniform awareness of children’s rights and needs will strengthen collective efforts to create an inclusive environment and support their optimal development.

Political analysis was conducted to obtain a view to group the actors’ power in determining the occurrence of something. Political analysis is intended to obtain a comprehensive picture of power and how to manage it. The discussion related to political analysis is presented in Table 2.

Political analysis is a crucial tool for understanding and managing various actors’ influence in achieving a goal or addressing a specific issue. In the context of empowering individuals or groups, five leading actors with distinct powers are outlined as follows (see Table 1):

- Regulators have the fundamental power to set policies and regulations. They determine direction through their ability to collaborate, control, lead and sustain initiatives. The strength of regulators makes them a key foundation in empowerment efforts, as they create a framework that can enable or limit other actors’ actions.
- As intellectual pillars, academics play a crucial role, possessing the power to produce and disseminate knowledge and ideas and engage in public discourse. Academics can also shape public opinion, encourage critical attitudes and advocate for public policy issues. The knowledge they produce serves as the foundation for innovation and evidence-based solutions.
- The community demonstrates operational and adaptive strength in advocacy, lobbying, mobilisation and empowerment. The community can also collaborate with external parties, provide information and education and be responsive to its members’ needs. They play a key role as agents of change at the grassroots level.
- Society and family hold the emotional and fundamental power of emotional bonds, care and affection. Family and surrounding community support form an essential psychological and social foundation for individual empowerment.
- Medical personnel possess practical expertise and direct connections in caring for individuals, especially those with disabilities; they serve as a bridge between families and the healthcare system. Their strength ensures the fulfilment of health needs and access to essential services.

**TABLE 1:** Social analysis of inclusive development for the resilience of children with cerebral palsy disabilities.

Actor	Value	Norm	Role
Regulator	Justice, equality and human rights	Ensuring accessibility and protection of the rights of persons with disabilities	Developing integrated policies and regulations that support inclusion
Academics	Knowledge, research and innovation	Developing an inclusive curriculum based on existing research	Educator, researcher and community contributor
Community	Community engagement, solidarity and empathy	Creating a friendly and supportive environment	Building a support network for persons with disabilities and advocating for their rights
Society or Family	Love, support and responsibility	Developing open and inclusive communication and collaboration	Providing emotional and financial support to family members with disabilities. Education support and cultural identity maintenance
Medical Personnel	Professionalism and development	Code of ethics, openness, transparency and active participation	Advocacy, education and training

**TABLE 2:** Political analysis in the development of inclusion for the resilience of children with cerebral palsy disabilities.

Actor	Strength
Regulator	Autonomy related to policies and regulations, cooperation, control, leadership and sustainability.
Academics	Knowledge, ideas and public discourse are produced and disseminated; public opinion and critical attitudes are formed and advocacy and campaigns on relevant public policy issues are conducted.
Community	Advocacy and lobbying, member mobilisation and empowerment, collaboration and empowerment, external party collaboration, information and education provision, responsiveness to members’ needs and participation in the policy process.
Society or Family	Emotional attachment, caring and loving.
Medical personnel	Direct experience in addressing the needs of people with disabilities can serve as a bridge between families and the service system.

Overall, synergistic management of the strengths of these actors is the key to success. Regulators provide legal and structural foundations; academics contribute data and theory; communities drive real actions on the ground; families offer moral and emotional support and medical professionals ensure physical well-being and access to services. The integration of these forces is essential for effective and sustainable empowerment.

The second phase of the soft systems approach delineates the issue scenario using rich visuals. At this point, relate the state and concerns of each involved stakeholder or entity. Rich visuals serve as a mechanism for articulating all interactions within the system (Lewis 1992). Marton and Booth (1996) asserted that rich graphics can facilitate many elements to provide a comprehensive explanation by combining information and processes while presenting options and inputs from each involved actor. Rich pictures serve as a versatile and global communication instrument for describing the state because they have no boundaries or limitations (Horan 2000).

All information obtained from experts is extracted into a rich picture. This is intended to describe the condition of resilience inclusion in children with CP disabilities. The preparation of a rich picture must be able to comprehensively describe the conditions that occur in an informative and easy-to-understand manner; in addition, ambiguity and misperception must be avoided. A rich picture is an important part of providing complete information to readers (Rittel & Webber 1973). This condition provides a challenge in the preparation process, requiring a process that is completely, accurately and communicatively prepared and avoids misperceptions (Berg & Pooley 2012; Checkland & Scholes 1991).

**FIGURE 1:**
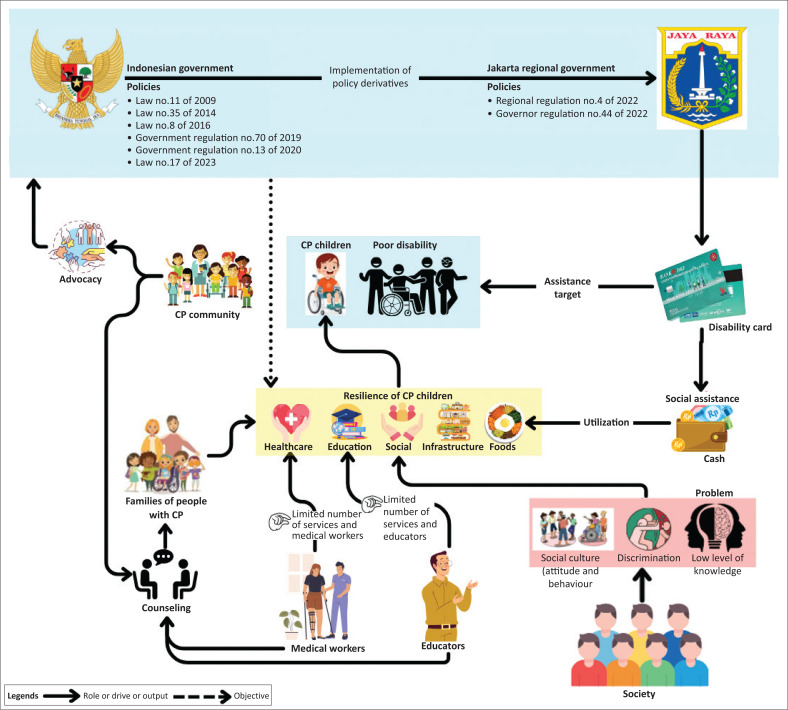
Rich picture conditions and disabilities.

Figure 1 illustrates how inclusion for resilience in children with CP is a way to achieve high-quality and empowered human resource excellence. The government wants to improve the quality of human resources and ensure that vulnerable and marginalised groups such as those with disabilities are not left behind. However, the policies so far have not been implemented and require genuine collaboration from all stakeholders to position disabilities not as objects of commercialisation but as resources with special abilities capable of high competitiveness.

There are several main actors in this resilience inclusion, namely (1) the government, (2) society, (3) communities, (4) medical personnel, and (5) inclusive educators, who require greater collaboration among them. The community is still rife with stigma and discrimination, as well as implementation that is not yet targeted and effective, and the number of medical and inclusive educators is still lacking. Figure 1 presents a full explanation.

The central and regional governments have tried to implement policies that support persons with disabilities, particularly children with CP. These efforts encompass various policies aimed at enhancing healthcare services, promoting inclusive education and fostering social empowerment for children with disabilities. Significant challenges remain in implementing these policies, considering that each stakeholder, whether it be the government, educational institutions, medical personnel, communities or families, has its roles and challenges in supporting the resilience of children with CP. The challenges faced in the development of inclusion for children with disabilities include policy inconsistencies. Although the government has implemented policies, their implementation is often not uniform across all regions, both at the central and local levels. This inconsistency is often caused by differences in understanding, lack of coordination between agencies and dependence on limited budgets. Weak data pose a significant obstacle to evidence-based decision-making. Inaccurate or inadequate data regarding the number of people with disabilities, their needs and the impact of existing policies lead to misguided and ineffective policies that fail to reach children with CP who require special attention.

Cross-sector collaboration among various stakeholders, such as the government, educational institutions, medical professionals, community organisations and the private sector, is needed. This collaboration is crucial for creating integrated solutions where each sector can contribute according to its specific competencies. The education sector can provide trained inclusive educators, whereas the health sector can improve the quality of medical services for children with disabilities. Cross-sector collaboration can also ensure that existing policies are more consistent and applied evenly across all regions.

Inclusive financial support must be part of the release strategy. People with disabilities, including children with CP, often face difficulties in accessing healthcare and education services because of financial constraints. The government must implement policies that facilitate access to more affordable services, whether through subsidies, health insurance or other forms of social assistance. In addition, the private sector and society can fund inclusive programmes that provide direct benefits to children with disabilities.

The next stage involves entering the system section. At this stage, a basic definition of building the inclusion of resilience of children with CP is obtained. At the bare stage, it aims to provide the following form: (1) Who and who will be included? (2) Who participates in the study? (3) Who can be affected and who can influence it? (Mehregan, Hosseinzadeh & Kazemi 2012). The exact verbal expression of a deliberate action system considered relevant to the investigation of the problem scenario is intended to ensure that the design is free from semantic defects; a basic definition needs a unique formula (Checkland & Scholes 1991).

In SSM, a formulation has been prepared that can be used to construct an understanding; in this case, the PQR formula (P through Q to reach R) can be used (Checkland & Scholes 1991). This understanding is called the root definition, which is then tested using CATWOE. The CATWOE concept is intended to test concepts in the root definition (Mathiassen & Nielsen 2000). Testing is intended to create a standard of findings on the issues that occur (Hardjosoekarto 2012). The root definition is as follows: Model of Inclusion for Children with Cerebral Palsy through Factor Grouping and Factor Integration to Enhance Resilience in Children with Cerebral Palsy.

The fundamental concept established in the advancement of disability inclusion for children with CP serves as a foundation for devising a developmental system capable of attaining the desired outcomes. All stakeholders must endorse the disability inclusion development system, which is fundamental for objectivity. Bergvall-Kareborn and Grahn (1996) evaluated the root concept by CATWOE analysis to emphasise objectivity. The findings are presented in Table 3.

**TABLE 3:** Customers, actors, transformation, worldview, owners, and environmental inclusion of resilience in children with cerebral palsy.

Element	Identification	Result
Customer	Who benefits from the change? Who is involved in the change process?	Persons with cerebral palsy, families, society, community, medical personnel, government and academics (educational institutions)
Actor	What changes do you want to see in the system?	Government, academics (educational institutions), community, family, society and medical personnel
Transformation	What are the expected significant picture changes?	Creating an inclusive ecosystem (social, economic and environmental) and integrated policies that impact the resilience of individuals with cerebral palsy
Worldview	The broad impact of the challenge	Increasing the resilience of individuals with CP and creating empowered disabilities towards the golden generation of 2045
Owner	Who owns the issue being researched?	Persons with cerebral palsy, families, society, community, medical personnel, government and academics (educational institutions)
Environment	What factors can influence the solution to the problem being studied?	Central and regional governments’ policies, databases, technology, human resources, infrastructure, social and inclusive economy

Collaboration between stakeholders is important. In this case, it is necessary to create a forum for regular collaboration that implements accountability for each party. Measurement is performed using 5E to obtain the expected information. The definition of expectations requires performance evaluation to ensure their suitability (Checkland & Scholes 1991). Experts emphasise that 5E-based measurement remains important in performance evaluation (Kotiadis et al. 2013). The results are presented in Table 4.

**TABLE 4:** 5E measurement to realise inclusion resilience in children with cerebral palsy.

Element	Information
Efficacy	The resilience of children with cerebral palsy disabilities can support the improvement of quality and human resources, especially for disabilities.
Efficiency	The increasing resilience and empowerment of individuals with cerebral palsy participating in various daily economic, social, cultural and political interactions.
Effectiveness	The stakeholder ecosystem is formed, and its goals are achieved through both resilience and business approaches.
Elegance	All stakeholders share the same view or perception to enhance the resilience of individuals with cerebral palsy.
Ethicality	The change process can be implemented and does not cause any harm to any party.

The fourth phase of the SSM involves conceptual model creation. Pereira et al. (2015) attempted to formulate a conceptual framework for the model. Checkland and Poulter (2006) explained that a conceptual model is based on the following:

- Sources from the root definition and CATWOE.
- Compiling several categories of activities, each of which is related to a specific change process.
- A series of activities can support parties in making adjustments and activities related to the entity conducting the transformation.

**FIGURE 2:**
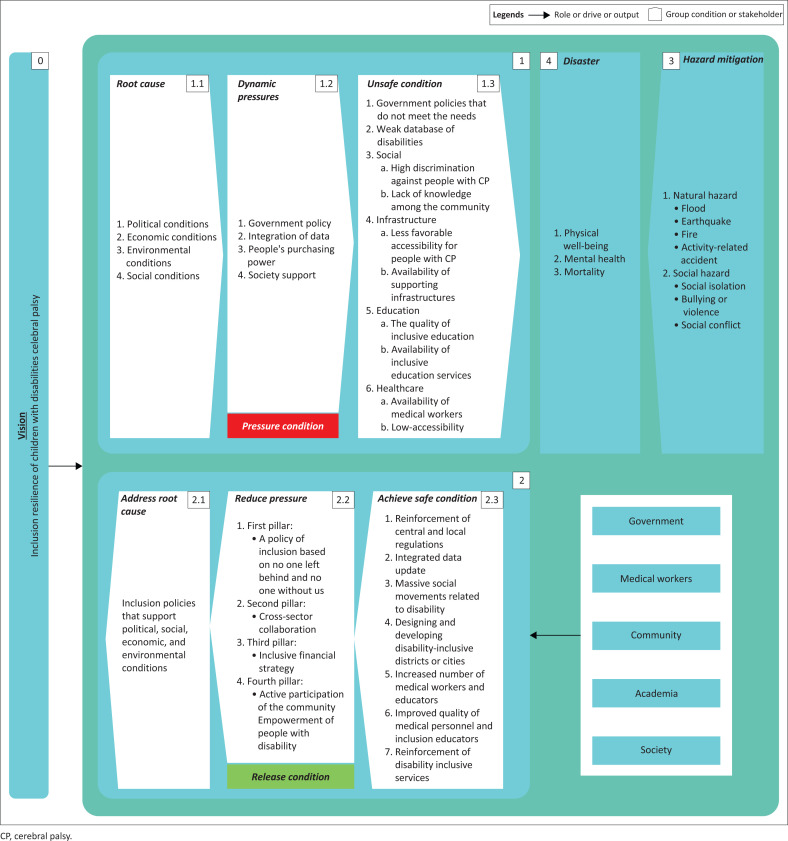
Conceptual model inclusion of resilience for children with cerebral palsy.

A conceptual model is a cognitive tool for analysing conditions and all world events (Hardjosoekarto 2012). The design must be appropriate to the context, descriptive and consistent with the desired system functionality (Fitriati 2015). Hidayat, Hubeis and Sukmawati (2020) outlined several key conceptual model parameters. Specifically, the conceptual model must be developed exclusively from terms contained in the basic definition, regardless of contextual relevance. If your conceptual model contains several activities or groups, these activities or groups need to be justifiably driven by terms. In preparation, choosing the right terms to describe the actions in the transformation process is necessary. This concept refers to the idea that activities in the conceptual models can be the basis for analysing related systems and formulating basic definitions of more complex conceptual models. (See Figure 2):

In the fifth phase of the soft system method, the conceptual model produced is compared with practical reality. The soft system method requires alternative perspectives to improve and modify the proposed model. Prasetyaningtyas (2019) explained that comparing models and their properties can stimulate discussions about perceptions and ultimately lead to beneficial changes to the model. When comparing conceptual models, it is important to identify key elements to reveal gaps in the model, such as (1) actors involved in the activity, (2) duration in carrying out the activity, (3) all activities that can be carried out, (4) opportunities for additions and adjustments, and (5) procedures for making improvements. (See Table 5):

**TABLE 5:** Real-world conditions with concepts in the resilience inclusion context for children with cerebral palsy.

No	Activity	Real world	Output	Reflection	Expert
0	Vision of creating CP Inclusion of Child Disability Resilience	The concept of CP Child Disability Resilience Inclusion as in UU No. 8 of 2016 is not as expected	Conceptualising the relationship between social, economic and environmental factors through the independence of children with cerebral palsy disabilities	Affirmation of CP Children with Disabilities Resilience Inclusion as a Dimension of National Resilience Inclusion using the highest legal umbrella	Brundtland 1987; Law of the Republic of Indonesia number 8 year 2016; Ungar et al. 2007
1	Pressure condition	Life pressure of children with cerebral palsy is very comprehensive	Identification of barriers and supports based on the prioritisation of problems in children with cerebral palsy	All parts of the pressure condition are described as a step in solving disability needs, challenges and problems	Adugna et al. 2020; Fernando et al. 2023; Lydall and Gerber 2021; Mbatha and Mokwena 2023
1.1	Root cause	Issues underlying resilience inclusion in political, social, economic and environmental conditions	Mapping the underlying political, social, economic and environmental conditions of this resilience inclusion issue	The need for a common perspective in understanding the needs, challenges and problems of individuals with disability	Delobel-Ayoub et al. 2022; Figueiredo et al. 2023; Selanon and Chuangchai 2023; Ullenhag et al. 2023; Wahyuningsi, Moita and Tanzil 2021
1.2	Dynamic pressure	Issues that people with cerebral palsy and their families find difficult to control	Mapping of political, social, economic and environmental problems	Vigilance of individuals with cerebral palsy and their families despite uncertainty	Adugna et al. 2020; Fernando et al. 2023; Lydall and Gerber 2021
1.3	Unsafe condition	Basic and close problems in daily life are still very much present and attention to children with CP who have not been maximised	Identifying and solving underlying problems in the field	Setting appropriate targets and indicators at each stage, namely, indicators related to resilience in the short, medium and long term	Gufron & Rahman 2020; Iwuagwu et al. 2023; Kusumaningrum et al. 2017; Smythe et al. 2024; Wahyuningsi et al. 2020
2	Release condition	Efforts to unravel the life stresses of children with cerebral palsy	Identification of barriers, de-stressing and support based on the prioritisation of problems in children with cerebral palsy	Mapping all internal and external elements used to unravel the life stress of children with cerebral palsy	Adugna et al. 2020; Fernando et al. 2023; Lydall and Gerber 2021
2.1	Address root cause	Support from the government, community and other stakeholders but still not forming inclusive policies	Government involvement is always present to assist in the formation of inclusive policies	Government commitment (central and local) to assist in the establishment of inclusive policies	Ahdiyana et al. 2021; Carty et al. 2021; Kappelides, Bould and Bigby 2021; Selanon and Chuangchai 2023; Ullenhag et al. 2023; Wiryawan 2022
2.2	Reduce pressure	A series of ways to reduce the stress of achieving inclusiveness in children with cerebral palsy	Map of important elements that play a role in reducing the stress of children with cerebral palsy	Identify and categorise elements that can play a role in reducing the stress of children with cerebral palsy	Adugna et al. 2020; Fernando et al. 2023; Lydall and Gerber 2021
2.3	Achieved a safe condition	The inclusive condition for children with cerebral palsy has not changed much to the positive even though it has been supported by various existing regulations.	Achieving an inclusive ecosystem that can support the needs of life as reflected in improved physical and mental health and reduced mortality	Mapping all elements that play a role in creating safe conditions to achieve inclusion for children with cerebral palsy	Gufron and Rahman 2020; Iwuagwu et al. 2023; Kusumaningrum et al. 2017; Smythe et al. 2024; Wahyuningsi et al. 2020
3	Hazard mitigation	Disaster mitigation efforts (natural and social) have been made but not in a broad spectrum	Disaster mitigation maps that can reach and be inclusive to increase resilience	Coordination based on academic studies as the basis for the disaster mitigation roadmap	Padhy 2021; Alviani and Asbara 2023; Westen 2013; Vadivelan et al. 2020
3.1	Natural hazard	Hazards of natural origin with great destructive power. Natural hazards (flood, earthquake, fire, activity accidents) can have uncertain probability and control	Design and development of inclusive areas in safe and disaster-prone environments	Collaboration with all stakeholders to design and build safe areas from natural disasters	Chen et al. 2022; Monforte et al. 2021; Mustika and Rahmayanti 2019; Padhy 2021; Putri 2024; Westen 2013
3.2	Social hazard	Social hazards were prevalent and still are. Social hazards are not physical but have psychological and mental impacts	Raising community social awareness to create an inclusive social environment	Campaigns and social movements aim to improve social inclusion	Gufron and Rahman 2020; Iwuagwu et al. 2023; Ostojic 2024; Wahyuningsi et al. 2020
4	Disaster	Complications of physical and mental health problems lead to high mortality rates	Improvements in physical and mental health and reduction in mortality in children with cerebral palsy	Design of an inclusion system that can increase the degree of resilience with physical, mental health and mortality indicators	MacEachern, Forkert and Dewey 2022; Sultana et al. 2022; Power 2020; Wouters, Evenhuis and Hilgenkamp 2020

CP, cerebral palsy.

There are two identifiable differences between the actual conditions and the conceptual model. (1) Conditions that do not exist in reality serve as an addition to the improvement of the concept being built. (2) Conditions that do not exist can frustrate researchers because they are unable to answer research questions and can encourage researchers to return to the second stage to collect new data and proceed to the next stage, namely detailed description, root definition and development of the conceptual model (Fitriati 2015). A different perspective is needed to improve the current situation in the world, which is a significant problem. Users of soft system methods face challenges in answering performance evaluation questions during the comparison phase (Checkland & Poulter 2006). The natural world’s complexity poses these challenges. Promoting diverse perspectives will increase the willingness to engage in radical action, leading to suggestions for change, improvement and refinement of real-world problems.

Framed as a resilience strategy, disaster mitigation encompasses proactive efforts to mitigate the risks and impacts of various hazards, both natural and social, to enhance the community’s capacity to recover and adapt. This concept extends beyond post-event response, focusing on preventing and reducing vulnerability before disasters occur. In the context of natural hazards, disaster mitigation includes the design and development of inclusive areas in environments that are safe from or vulnerable to disasters. This involves collaboration with all stakeholders to design and build facilities that are resilient to threats such as floods, earthquakes and fires. The goal is to minimise physical damage and casualties while ensuring accessibility for all individuals, including those with disabilities, during and after a disaster.

Social hazard mitigation focuses on raising community social awareness to create an inclusive social environment. This involves large-scale campaigns and social movements aimed at increasing social inclusion and mitigating the impact of non-physical hazards, such as discrimination, negative stigma or lack of knowledge, which can have significant psychological and mental health impacts on vulnerable groups. Thus, disaster mitigation as a resilience strategy is not only oriented towards physical protection but also towards strengthening social cohesion and the adaptive mentality of society as a whole.

## Conclusion

The development of inclusion for the resilience of children with CP disabilities requires strategic approaches and steps that can achieve high-quality and empowered human resource excellence. In this context, the alignment of concepts and future views is essential for the collaboration between stakeholders. This condition allows stakeholders to work more effectively and efficiently to implement resilience inclusion for children with CP. On the other hand, stakeholders need to know in detail and dare to choose the factors that must be promoted to implement resilience inclusion for children with CP disabilities. Currently, stakeholders are required to engage in open communication and build trust among parties to view disabilities as resources with special abilities and high competitiveness. The roadmap is a crucial element in the implementation of resilience inclusion for children with CP, so that they can have an appropriate plan based on the time frame (short term, medium term, and long term). The implementation process requires specific and attractive policies to support the development of inclusion in improving its implementation. Specific and attractive policies are needed to support the development of inclusion in improving the resilience of children with CP. The inclusion of resilience in children with CP is inseparable from data updates that can be integrated to ensure proper and targeted implementation. Strengthening inclusive services in various sectors must be implemented comprehensively and prioritised to achieve equity in the quality and quantity of inclusive medical personnel and educators. Finally, a broad social movement related to education and the equitable distribution of understanding is a crucial aspect that must be considered to reduce stigma and discrimination, as well as to encourage all parties to design and build inclusive areas that are friendly to disabilities.
